# A Possible Role for CD8+ T Lymphocytes in the Cell-Mediated Pathogenesis of Pemphigus Vulgaris

**DOI:** 10.1155/2013/764290

**Published:** 2013-11-18

**Authors:** Federica Giurdanella, Luca Fania, Maria Gnarra, Paola Toto, Daniela Di Rollo, Daniel N. Sauder, Claudio Feliciani

**Affiliations:** ^1^Department of Dermatology, Policlinico A. Gemelli Hospital, Catholic University of the Sacred Heart, Largo Agostino Gemelli 8, 00168 Rome, Italy; ^2^Department of Dermatology, University G. D'Annunzio of Chieti-Pescara, Via dei Vestini 5, 66013 Chieti, Italy; ^3^Department of Medicine and Aging Science, University G. D'Annunzio of Chieti-Pescara, Via dei Vestini 31, 66013 Chieti, Italy; ^4^Department of Dermatology, Princeton University Hospital, 253 Witherspoon Street, Princeton, NJ 08540, USA; ^5^Faculty of Medicine, University of Ottawa, 451 Smyth Rd., Ottawa, ON, Canada K1H8M5

## Abstract

Pemphigus vulgaris (PV) is an autoimmune blistering disease whose pathogenesis involves both humoral and cell-mediated immune response. Though the pathogenetic role of autoantibodies directed against desmoglein 3 is certain, a number of other factors have been suggested to determine acantholysis in PV. In this study we examined the possible role of CD8+ T cells in the development of acantholysis by a passive transfer of PV autoantibodies using CD8 deficient mice, and we also studied the inflammatory infiltrate of PV skin lesions by immunohistochemical staining. The results of the immunohistochemical staining to study the expression of CD3, CD4, and CD8 in PV skin lesions showed that CD4+ are more expressed than CD8+ in the inflammatory infiltrate of PV lesions, confirming the data of the previous literature. The passive transfer study showed a lower incidence of pemphigus in the group of CD8 deficient mice compared to the control one of wild-type mice. These results suggest that CD8+ T cells may play a role in the pathogenesis of PV, perhaps through the Fas/FasL pathway.

## 1. Introduction

Pemphigus vulgaris (PV) is a life-threatening autoimmune blistering disease mediated by autoantibodies (autoAbs) directed against desmogleins (Dsg) located on the surface of keratinocyte cells (KC). This leads to an intraepithelial loss of adhesion called acantholysis, and clinically it presents with vescicles and blisters [1]. AutoAbs in PV are directed mainly against desmoglein 3 (Dsg 3), a desmosomal glycoprotein situated in the skin predominantly in the suprabasilar epidermal layer, and less frequently against desmoglein 1 [2]. Though the pathogenetic role of antidesmoglein autoAbs is certain, the exact mechanism through which they lead to acantholysis is still incompletely understood. Complement [3], plasminogen-plasmin [4], cytokines [5], cell-mediated immunity, and other autoantibodies such as anticholinergic receptor antibodies have been suggested in determining acantholysis in PV [6]. Studies conducted so far regarding the role of T cells involved mainly CD4+ lymphocytes for their cooperation with B cells and subsequently for the induction and regulation of autoAbs production [7]. The function of CD8+ T cells has not been explored yet, but some authors hypothesize their role in cell-mediated pathogenesis of PV [8]. Other studies suggested a possible role of natural killer (NK) cells [9] as well as Fas and caspase 8 in PV [10]. These molecules' function in the apoptosis mechanism is well known. In PV these molecules result in a shrinking of keratinocytes that leads to detachment inducing acantholysis [11]. Fas is a member of the tumor necrosis factor (TNF) receptor family that is bound by Fas ligand, expressed on T CD8+ cells. In this study we sought to evaluate the role of CD8+ cells performing a passive transfer of PV autoAbs using CD8 deficient mice (CD8^−^/^−^). The results of these studies suggest a role for CD8 in the pathogenesis of PV.

## 2. Materials and Methods

### 2.1. Immunohistochemistry

Immunohistochemical staining was performed using the alkaline phosphatase-antialkaline phosphatase (APAAP) method on 7 *μ*m skin sections of 7 PV patients [12]. Monoclonal antibodies against CD3 (1 : 20; DAKO, Glostrup, Denmark), CD4 (1 : 20; DAKO), and CD8 (1 : 20; DAKO) were used. For the quantitative study, stained cells were first counted in three consecutive microscopic fields (250x), both in the dermis and in the epidermis, and then summed; the average value was then calculated.

### 2.2. Preparation of Pemphigus IgG

Plasma was obtained from the plasmapheresis of one patient with clinical, histologic, and immunologic features consisting of the diagnosis of PV, during the acute phase of the disease. Total IgG concentration was measured by nephelometry using monospecific goat anti-human IgG (Beckman Instruments, Missasauga, ON, Canada). Pemphigus Ab titers were measured by indirect immunofluorescence (IIF) using monkey esophagus epithelium as the tissue substrate [13]. As a negative control, IgG fractions were isolated and removed from PV plasma using protein A (PA). Isolation of IgG fractions from PV plasma was achieved by standardized technique using staphylococcal protein A coupled to Sepharose 4B [14] (Pharmacia Biotech, Uppsala, Sweden). PA was washed four times in cold PBS and finally incubated with PV plasma overnight at 4°C. The supernatant was collected and used as negative control. Absence of IgG fractions in the control plasma was assessed by IIF staining on a monkey esophagus epithelium substrate and confirmed by nephelometry. PV plasma and control plasma were filter sterilized with Millex (pore size 0.22 mm; Millipore, Bedford, MA) and stored at −20°C.

### 2.3. Mice

The following strains were used: CD8^−^/^−^ and C57BL/6 (CD8^−^/^−^ control). All mice were housed and bred under specific pathogen-free conditions in the animal facility of the Sunnybrook Health Science Centre. Neonates (<24 h of age) were used. An average of 15 mice within each experimental group was used, and each experiment was repeated at least three times. All animal procedures were approved by the Sunnybrook Health Science Centre animal care committee.

*CD8* ^−^ */* ^−^ *Mice.* The generation of mice homozygous for CD8 gene mutations (CD8^−^/^−^) was obtained by disruption of the Lyt-2 gene through homologous recombination, and the mutation was interbred into the C57BL/6 background before generating CD8-deficient (CD8^−^/^−^) mice. Mice homozygous for the defect were used as the knockout (KO) mice, with the wild-type (WT) animals serving as the nondeficient controls.

### 2.4. Passive Transfer Model

To induce PV in mice, we utilized the model of Anhalt et al. [15] with minor modifications. Briefly, plasma was injected intradermally, in the dorsal area, into neonatal mice through a 30-gauge needle. The total dose administered ranged from 30 to 50 *μ*L/g of body weight in a single administration. We chose a dose of 30 *μ*L/g because this was the minimum dose inducing the disease in WT mice. CD8^−^/^−^ mice and C57BL/6 mice were injected with the same dose of PV plasma. As a negative control, gene targeted mutant mice and WT mice were injected with plasma depleted of IgG by treatment with protein A. Mice were examined 24 h after the injections. Cutaneous lesions consisting of intact blisters or erosions were enumerated.

### 2.5. Tissue Specimens Staining

Lesional and perilesional skin were obtained for light microscopy and direct immunofluorescence (DIF) 24 h after injection with PV IgG. At the time of biopsies, serum was also obtained and assayed for IIF on a monkey esophagus epithelium to detect the IgG titer.

### 2.6. Direct Immunofluorescence

Perilesional skin was biopsied and specimens were snap frozen in liquid nitrogen until use. Cryostat sections (5 *μ*m) were used, and DIF studies were performed according to standard techniques [16]. Briefly, specimens were washed in PBS for 10 minutes, incubated for 30 minutes with FITC-conjugated F(ab′)_2_ fragment of rabbit anti-human IgG, specific for *γ*-chains (1 : 25; Dako, Glostrup, Denmark), and washed in PBS for 15 minutes. Slides were covered with buffered glycerol, and results were read in a Nikon Optiphot immunofluorescence microscope (Nikon, Melville, NY).

### 2.7. Indirect Immunofluorescence

Sera were collected 24 h after PV IgG treatment or PA treatment (IgG depleted), and IIF studies were performed according to standard techniques [16]. Cryostat sections (5 *μ*m) of monkey esophagus were used as substrate, washed for 10 min in PBS, incubated for 30 min with different concentrations of sera (1 : 1-1 : 600), washed in PBS for 15 min, labeled with FITC-conjugated F(ab′)_2_ fragment of rabbit anti-human IgG (DAKO, Glostrup, Denmark) for 30 min, and then washed again in PBS for 10 min. Slides were covered with buffered glycerol, and results were examined using a Nikon Optiphot immunofluorescence microscope (Nikon, Melville, NY).

### 2.8. Histologic Technique

Skin biopsies from mice were fixed in 10% formalin and stained with hematoxylin and eosin.

### 2.9. Statistical Analysis

Data regarding the incidence of the disease in KO and control mice were analyzed using the *χ* ^2^ test; a *P* value <0.05 was considered to be significant.

## 3. Results

### 3.1. Immunohistochemistry CD3-CD4-CD8

CD3+ T cells were detected both in the dermis (32.8 ± 1.6 cells counted as mentioned in Section 2), with a perivascular distribution, and in the epidermis (7.9 ± 2.8) of all patients. CD4+ T cells were found in the superficial and papillary dermis (33.6 ± 4.8) with scattered and perivascular distribution, and a fewer number of them were detected in the basal and suprabasal layers near the dermal-epidermal junction (4.2 ± 1.2). A number of CD8+ T cells (14.2 ± 1.6) were observed in perivascular areas of dermal lesional skin (CD4/CD8 = 2.7) (Table 1).

### 3.2. Pemphigus Vulgaris IgG

Pemphigus plasma was obtained as described in Section 2. IIF for PV IgG using monkey esophagus as substrate demonstrated a titer of 1 : 2460, and an IgG concentration of 5.9 mg/mL was measured by nephelometry. PA-treated plasma showed absence of intercellular staining on monkey esophagus, and IgG levels were below the level of detection using the nephelometric analysis.

### 3.3. Passive Transfer of PV

The passive transfer of PV IgG demonstrated a direct correlation between the amount of PV IgG injected and the incidence of the disease. Acantholysis and inflammatory infiltrate were evident in mice given plasma containing PV IgG and absent in mice injected with IgG-depleted plasma. The epidermis of mice injected with PV IgG who developed PV lesions showed human IgG bound to the intercellular cell surface by DIF. No staining was found in mice injected with PA-treated plasma. No difference was observed in the intensity of fluorescence in different strains of mice treated with an equal dose of PV IgG. The IgG titer in all PV plasma injected mice detected by IIF ranged between 1 : 100 in mice treated with 30 *μ*L/g PV plasma and 1 : 200 in mice injected with 50 *μ*L/g. No circulating PV IgG were detected in mice injected with PA-treated plasma. In all of the WT mice, IgG deposits were observed with a minimal dose of 30 *μ*L/g PV plasma (177 *μ*g/g PV IgG). At this dose, about 10% of mice displayed clinical evidence of disease. With an administered dose of 50 *μ*L/g PV plasma (295 *μ*g/g PV IgG), 75% of the WT mice developed blisters.

When a dose-response study was performed on CD8^−^/^−^ mice, a lower incidence of pemphigus was observed. In particular, with a dose of 30 *μ*L/g PV plasma, 0% (0/5) of the CD8^−^/^−^ mice injected showed evidence of disease as compared with 9.5% (4/42) of C57BL/6 mice (difference statistically not significant). With an injected dose of 50 *μ*L/g, 44% (13/29) of the CD8^−^/^−^ mice developed PV lesions, compared with 72% (26/36) of the control group (difference statistically significant) (Figure 2). 

## 4. Discussion and Conclusions

The immunopathogenesis of PV involves both humoral and cell-mediated response. While antibodies to desmoglein are pathogenic, CD4+, CD8+, and NK cells are also implicated. The function of CD4+ cells has previously been examined. It is known that CD4+ Th1 cells promote the IgG1 production by means of B cells, while IgG4, IgA, and IgE autoAbs are induced by the cooperation of CD4+ Th2 with B cells [9]. The function of CD8+ cells in the pathogenesis of PV is still unclear, but their role has been hypothesized by some authors. They occasionally observed that CD8+ T cells from patients with active PV were responsive to in vitro stimulation with Dsg 3. Specifically they secreted IL-2 and INF-*γ* but not IL-4 or IL-5, being the first cytokines implicated in the cell-mediated immune response and the latters in the humoral one. This suggests a role for CD8+ T cells in the cell-mediated pathogenesis of PV [9].

**Figure 1:**
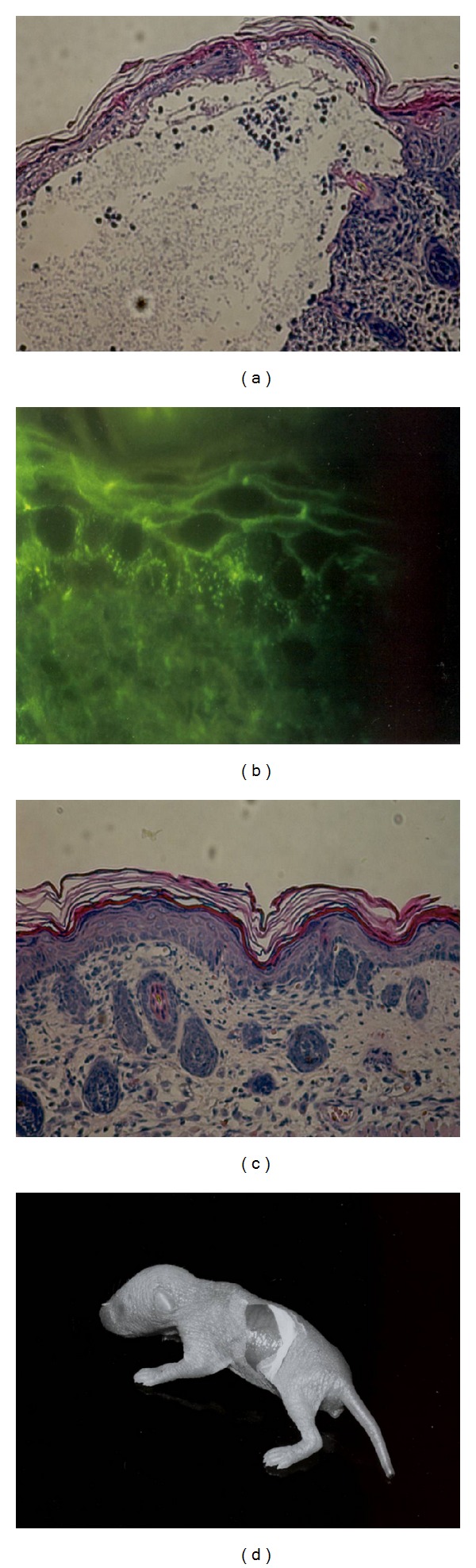
(a) Histology of a PV lesion from a wild-type mice injected with PV IgG. (b) Direct immunofluorescence in wild-type mice injected with PV IgG showing IgG bounded to the intercellular cell surface (100x). (c) Histology of mice treated with IgG deprived plasma, no acantholysis is observed. Immunofluorescence studies were also negative (data not shown). (d) A large erosion on the back of a mice treated with PV IgG.

**Figure 2:**
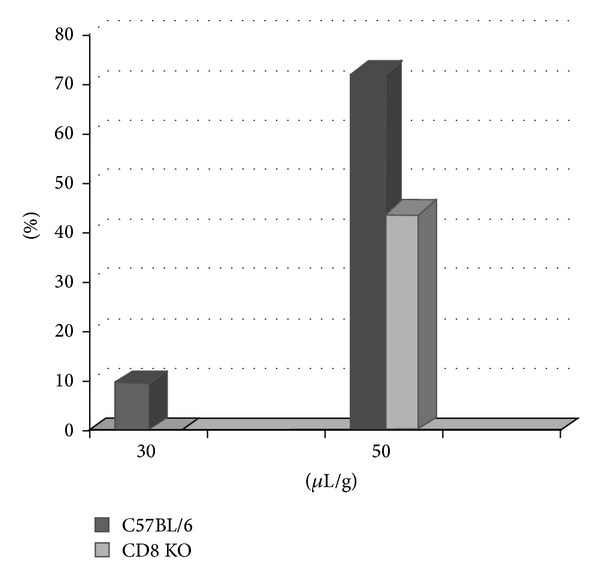
Incidence of disease in KO mice and control-C57BL/6 mice in passive transfer model of IgG PV. With a dose of 30 *μ*L/g PV plasma, 0% (0/5) of the CD8^−^/^−^ mice developed PV, compared with 9.5% (4/42) of C57BL/6 mice. With an injected dose of 50 *μ*L/g, 44% (13/29) of the CD8^−^/^−^ mice showed PV lesions, compared with 72% (26/36) of the control group.

The characterization of the infiltrate of PV human skin lesions showed mostly CD4+ T-cells and less commonly, about 10%, CD8+ cells [17]. In our study we analyzed the T cell infiltrate of skin lesions in patients affected by PV by means of immunohistochemistry for the expression of CD3, CD4, and CD8. The results confirm the data of the literature because CD4+ are more expressed in PV human skin lesions than CD8+ with a ratio of 2.7. Control mice (WT) developed PV lesions (9.5% with 30 *μ*L/g and 72% with 50 *μ*L/g), whereas CD8 knockout group was relatively resistant (0% with 30 *μ*L/g and 44% with 50 *μ*L/g). As expected, mice that received 50 *μ*L/g PV plasma developed more PV lesions than those injected with 30 *μ*L/g. Histological analyses and DIF and IIF tests confirmed the diagnosis of PV in mice that presented cutaneous lesions after the injection of PV plasma, while in the totality of mice treated with IgG deprived plasma, all tests were negative (Figure 1). These results suggest that CD8+ T cells may play a role in the pathogenesis of PV. CD8+ T cells mediate immunity in part through granzymes or inducing apoptosis by the Fas/Fas ligand (FasL) system. This death cell pathway depends mainly on Fas/FasL interaction through the activation of the caspases system. Some reports showed that, in keratinocytes treated with pemphigus sera, the activation of caspases was observed. Other reports demonstrated that inhibitors of caspases provoke the blockade of acantholysis [11]. An in vitro study showed that the addiction of anti-FasL Abs has partially inhibited the IgG-PV induced apoptosis in cultures of keratinocytes [11].

We thus suggest a role of CD8+ T lymphocytes in the pathogenesis of PV which in part may be mediated through perhaps Fas-FasL signaling. 

## Figures and Tables

**Table 1:** T cellular markers identified in human skin lesions of PV patients by immunohistochemistry. The calculated average number of stained cells in three consecutive microscopic fields (250x) is reported.

	Dermis	Epidermis
CD3	32.8 ± 1.6	7.9 ± 2.8
CD4	33.6 ± 4.8	4.2 ± 1.2
CD8	14.2 ± 1.6	2.9 ± 1.3
